# London Prices of Drugs.—Jan. 28

**Published:** 1814-02

**Authors:** 


### LONDON PRICES OF DRUGS.—JAN. 28.

*(To be continued regularly.)*

| | £. | s. | d. | £. | s. | d. per |
|---|---|---|---|---|---|---|
| Aloes Barbadoes, from | 20 | 10 | 0 to | 21 | 0 | 0 C. |
| ——Cape | 10 | 0 | 0·· | 11 | 0 | 0 — |
| ——Succotrina | 20 | 0 | 0·· | 0 | 0 | 0 — |
| ——Epatica or E.I. | 10 | 0 | 0·· | 12 | 0 | 0 — |
| Angelica Root | 12 | 0 | 0·· | 13 | 0 | 0 — |
| Alkanet Root | 7 | 0 | 0·· | 8 | 0 | 0 — |
| Antimony Crude | 3 | 0 | 0·· | 3 | 5 | 0 — |
| Aqua Fortis | S. | 0 | 8··D. | 1 | 1 | lb |
| Arrow Root fine | 0 | 2 | 0·· | 0 | 3 | 6 — |
| ——ordinary | 0 | 1 | 0·· | 0 | 1 | 6 — |
| Arsenic Red | 6 | 0 | 0·· | 6 | 6 | 0 C. |
| ——White | 3 | 10 | 0·· | 3 | 12 | 0 — |
| Balsam Capivi | 0 | 5 | 6·· | 0 | 0 | 0 lb. |
| ——Peru | 0 | 19 | 0·· | 1 | 0 | 0 — |
| ——Tolu | 0 | 12 | 0·· | 0 | 13 | 0 — |
| Bark Jesuits Com. | 0 | 3 | 6·· | 0 | 0 | 0 — |
| ——Second | 0 | 6 | 0·· | 0 | 7 | 0 — |
| ——Quil or best | 0 | 8 | 0·· | 0 | 10 | 0 — |
| ——Red | 0 | 7 | 0·· | 0 | 10 | 0 — |
| ——Yellow | 0 | 4 | 6·· | 0 | 5 | 0 — |
| Borax refined E.I. | none. | | | | | C. |
| ——English | 0 | 3 | 6·· | 0 | 3 | 8 lb. |
| ——unrefined or Tinc. | 10 | 0 | ·· | 11 | 0 | 0 C. |
| Camphire refined | 0 | 8 | 9·· | 0 | 9 | 0 lb. |
| ——unrefined | 28 | 0 | 0·· | 32 | 0 | 0 C. |
| Cantharides | 0 | 10 | 6·· | 0 | 11 | 0 — |
| Cardamoms (best) | 0 | 7 | 6·· | 0 | 0 | 0 lb. |
| Cassia Buds | 12 | 0 | 0·· } | | 0 | 0 C. |
| ——Fistula W.I. | 12 | 0 | 0·· } in bond | | | — |
| ——Lignea | 12 | 0 | 0·· } | | 0 | 0 — |
| Castoreum American | 2 | 10 | 0·· | 2 | 15 | 0 lb. |
| ——Russia | 11 | 0 | 0·· | 0 | 0 | 0 — |
| Castor Oil per bottle 1½ lb. | 0 | 3 | 9·· | 0 | 4 | 3 bo |
| Cocculus Indicus | 3 | 0 | 0·· | 0 | 0 | 0 C. |
| Colocynth Turkey | 0 | 5 | 0·· | 0 | 5 | 6 lb. |
| Columbo Root | 2 | 15 | 0·· | 3 | 0 | 0 C. |
| Cream of Tartar | 7 | 10 | 0·· | 9 | 0 | 0 — |
| Gallangal East-India | 5 | 0 | 0·· | 0 | 0 | 0 C. |
| Gentian Root | 3 | 15 | 0·· | 4 | 0 | 0 — |
| Ginseng | 0 | 1 | 6·· | 0 | 0 | 0 lb. |
| Grains of Guinea | 5 | 10 | 0·· | 6 | 0 | 0 C. |
| Gum Ammo. Drop | 30 | 0 | 0·· | 0 | 0 | 0 — |
| ——Lump | 8 | 0 | 0·· | 9 | 0 | 0 — |
| ——Animi | 3 | 10 | 0·· | 7 | 10 | 0 — |
| ——Arabic E.I. | 2 | 0 | 0·· | 4 | 0 | 0 — |
| ——Turkey Fine | 8 | 10 | 0·· | 9 | 9 | 0 — |
| ——Barbary | 6 | 0 | 0·· | 7 | 0 | 0 — |
| ——Assafœtida | 10 | 0 | 0·· | 15 | 0 | 0 — |
| ——Benjamin | 8 | 10 | 0·· | 35 | 0 | 0 — |
| ——Cambogium | 20 | 0 | 0·· | 26 | 0 | 0 — |
| ——Copal scraped | 0 | 2 | 0·· | 0 | 3 | 6 lb. |
| ——Galbanum | 16 | 0 | 0·· | 20 | 0 | 0 C. |

| | £. | s. | d. | £. | s. | d. per |
|---|---|---|---|---|---|---|
| Gum Guaiacum, from | 0 | 2 | 6 to | 0 | 3 | 0 lb. |
| ——Mastic | 0 | 4 | 6·· | 0 | 4 | 9 — |
| ——Myrrh | 15 | 0 | 0·· | 18 | 0 | 0 C. |
| ——Olibanum | 6 | 0 | 0·· | 7 | 0 | 0 — |
| ——Oppoponax | 80 | 0 | 0·· | 0 | 0 | 0 — |
| ——Sandrac | 6 | 10 | 0·· | 8 | 0 | 0 — |
| ——Seneca Garbled | 5 | 2 | 0·· | 5 | 5 | 0 — |
| ——Tragacanth | 36 | 0 | 0·· | 38 | 0 | 0 — |
| Jalap | 0 | 5 | 0·· | 0 | 5 | 3 lb. |
| Ipecacuanha | 0 | 19 | 0·· | 1 | 0 | 0 — |
| Isinglass Book | 0 | 10 | 6·· | 0 | 11 | 0 — |
| ——Leaf | 0 | 10 | 6·· | 0 | 11 | 0 — |
| ——Long Staple | 0 | 10 | 6·· | 0 | 11 | 0 — |
| ——Short Staple | 0 | 10 | 6·· | 0 | 11 | 0 — |
| Manna Flakey | 0 | 6 | 0·· | 0 | 6 | 6 — |
| ——Sicily in Sorts | 0 | 4 | 0·· | 0 | 5 | 6 — |
| Musk China | 0 | 18 | 0·· | 1 | 0 | 0 oz. |
| Nux Vomica | 3 | 0 | 0·· | 3 | 10 | 0 C. |
| Oil of Vitriol | 0 | 0 | 3¾ | 0 | 0 | 0 lb. |
| Opium East-India | 0 | 19 | 6·· | 1 | 0 | 0 — |
| ——Turkey | 1 | 9 | 0·· | 1 | 10 | 0 — |
| Pink Root | 0 | 4 | 0·· | 0 | 4 | 6 — |
| Quicksilver | 0 | 4 | 10·· | 0 | 0 | 0 — |
| Rhubarb East-India | 0 | 3 | 0·· | 0 | 5 | 0 — |
| ——Russia | 0 | 18 | 0·· | 1 | 0 | 0 — |
| Saffron Spanish | 2 | 5 | 0·· | 2 | 10 | 0 — |
| ——French | 1 | 15 | 0·· | 2 | 0 | 0 — |
| Sago | 6 | 0 | 0·· | 6 | 10 | 0 C. |
| Sal. Ammoniac | 10 | 10 | 0·· | 11 | 0 | 0 — |
| Sarsaparilla | 0 | 3 | 0·· | 0 | 3 | 6 lb. |
| Sassafras | 8 | 0 | 0·· | 9 | 0 | 0 T. |
| Scammony Aleppo | 1 | 6 | 0·· | 1 | 7 | 0 lb. |
| ——Smyrna | 0 | 10 | 0·· | 0 | 12 | 0 — |
| Senna | 0 | 3 | 0·· | 0 | 0 | 0 — |
| Seeds Anni Alicant | 6 | 0 | 0·· | 6 | 10 | 0 C. |
| ——Coriander English | 1 | 10 | 0·· | 1 | 16 | 0 — |
| ——Cummin | 7 | 0 | 0·· | 7 | 10 | 0 — |
| ——Fenugreek | 1 | 10 | 0·· | 1 | 13 | 0 — |
| Shellack | 7 | 0 | 0·· | 8 | 8 | 0 — |
| Sticklack | 6 | 0 | 0·· | 8 | 0 | 0 — |
| Snake Root | 0 | 0 | 0·· | 0 | 0 | 0 lb. |
| Soap Castile or Spanish | 12 | 0 | 0·· | 14 | 0 | 0 C. |
| Spermaceti refined | 0 | 2 | 7·· | 0 | 0 | 0 lb. |
| Tamarinds West-India | 8 | 10 | 0·· | 9 | 0 | 0 C. |
| Tapioca Lisbon | 0 | 0 | 8·· | 0 | 1 | 0 lb. |
| Turmeric Bengal | 3 | 3 | 0·· | 0 | 0 | 0 C. |
| ——China | 4 | 5 | 0·· | 4 | 15 | 0 — |
| ——West-India | 8 | 0 | 0·· | 9 | 0 | 0 — |
| Verdigris Wet | 0 | 3 | 6·· | 0 | 0 | 0 lb. |
| ——Dry | 0 | 5 | 8·· | 5 | 10 | 0 — |
| ——Crystallized | 0 | 8 | 6·· | 0 | 9 | 0 — |
| Vitriol Roman | 0 | 0 | 7·· | 0 | 0 | 0 — |
| ——Foreign white | 3 | 16 | 0·· | 4 | 0 | 0 C. |

*Price of Vials per Gross.*—8 oz. 70s.—6 oz. 58s.—4 oz. 47s.—3 oz. 43s.—2 oz. 36s.—1 oz. 30s.—½ oz. 24s.

### OBSERVATIONS.

At recent Sales of Merchandize by Auction, *Messrs.* PISTOR *and* Co. sold 10 Casks of Gum Arabic, 7l. 5s. to 7l. 14s. per Cwt.—1 Cask Tamarinds, 9l. 6s. per Cwt.—1 Keg Castorum, 42s. 6d. per lb.—24 Chests Rough Camphire, 28l. 15s. to 29l. per Cwt.—14 Chests Jesuits' Bark, 2s. to 7s. 3d. per lb.—9 Barrels Gum Guaiacum, 10d. to 12d. per lb.—145 Serons Carthagena Bark, 7d. to 20d. per lb.——Messrs. BOWDEN and TUCKER sold 82 Chests Pale Peruvia Bark, bonded, 3s. 8d. to 8s. 3d. per lb.—26 Bales Gentian, 73s. to 74s. per Cwt.—5 Chests Rhubarb, duty paid, 9s. 2d. to 9s. 6d. per lb.—2 Chests Gum Ammoniac, 7l. 10s to 8l. 5s. per C

